# Hypoxia-inducible factor 1αa regulates lipid metabolism to coordinate adipocyte hypertrophy and hyperplasia in grass carp

**DOI:** 10.1016/j.jbc.2026.111195

**Published:** 2026-01-27

**Authors:** Mingkui Wei, Zhiqi Tian, Lei Song, Rongrong Xue, Handong Li, Hong Ji, Jian Sun

**Affiliations:** College of Animal Science and Technology, Northwest A&F University, Yangling, China

**Keywords:** adipocyte hyperplasia, adipocyte hypertrophy, HIF1αa, ATGL, PPARγ

## Abstract

Obesity significantly burdens global health. Conversely, some animals efficiently store substantial fat during food abundance while maintaining metabolic health, offering unique insights into the mechanisms of healthy adipose expansion. Understanding these short-term physiological adaptations is therefore crucial. Here, using the grass carp (*Ctenopharyngodon idellus*) as a model, which exhibits remarkable fat storage capacity, we found that adipose tissue responded to energy overload first and expanded through adipocyte hypertrophy and hyperplasia. Mechanistically, hypoxia inducible factor 1αa (HIF1αa) was activated in mature adipocytes after short-term high-energy intake, thereby bidirectionally regulating adipose triglyceride lipase (ATGL) to drive healthy expansion of adipose tissue in grass carp: (1) HIF1αa downregulates ATGL protein levels *via* the ubiquitin-proteasome pathway, promoting adipocyte hypertrophy; (2) HIF1αa upregulates ATGL transcription to sustain basal lipolysis, releasing free fatty acids that activate peroxisome proliferator–activated receptor γ in preadipocytes to promote adipocyte hyperplasia. Crucially, unlike obese mice requiring 7 weeks, grass carp exhibited rapid adipocyte hyperplasia. This not only increases the energy storage limit but also prevents excessive hypertrophy of adipocytes. Taken together, our study reveals how grass carp utilizes hypoxia signal (a signal often associated with metabolic disorders in mammals) to coordinate the pattern of adipose tissue expansion, achieving rapid and healthy lipid storage. Our findings redefine hypoxia's role as a metabolic orchestrator rather than a stress indicator, providing a theoretical basis for addressing obesity-related diseases in humans caused by excessive energy intake.

Food scarcity poses a significant challenge for animals in nature (1, 2). To survive periods of limited resources, diverse species have evolved unique adaptive strategies (1). Among these, a key approach involves the storage of substantial energy reserves in short term during times of abundance for food scarcity (1). Lipids, due to their high energy density and excellent stability, represent the optimal substrate for long-term energy storage in animals (3, 4, 5). This strategy is exemplified by species such as grizzly bears, arctic ground squirrels, and fish, which accumulate significant fat to endure extended periods of nutritional deprivation (2, 6, 7, 8). However, in contrast, an excessive accumulation of lipids in humans is accompanied by chronic tissue inflammation and the onset of metabolic diseases (9, 10). Therefore, deciphering the physiological mechanisms by which these animals efficiently and safely store large amounts of energy can provide insights for the prevention and management of obesity and related metabolic diseases caused by overnutrition in humans.

Central to the ability to safely manage short-term energy surplus is the expansion of adipose tissue, the primary organ for lipid storage (11). When the expansion capacity of adipose tissue fails to adequately accommodate excess energy intake, ectopic lipid deposition and metabolic disorders ensue (12). Adipose tissue primarily expands to store energy through adipocyte hyperplasia (preadipocytes proliferate and differentiate into new adipocytes) and adipocyte hypertrophy (enlargement of lipid droplets in existing mature adipocytes) (9, 13). Studies in obese mouse models and obese human subjects demonstrate that adipocyte hyperplasia was impaired, leading to excessive adipocyte hypertrophy and consequent tissue inflammation (13, 14). This evidence strongly suggests that adipocyte hypertrophy represents a less sustainable strategy for energy storage, whereas adipocyte hyperplasia is crucial for achieving substantial and safe lipid accumulation (15, 16). Studies on mice have revealed that the generation of new adipocytes takes approximately 7 weeks (17, 18, 19). These findings collectively indicate that the insufficient capacity for adipocyte hyperplasia underlies the inability of mouse and human to safely store large amounts of energy in the short term. However, it remains unclear how the processes of adipocyte hyperplasia and hypertrophy are orchestrated in animals uniquely adapted to short-term high-energy intake, enabling their efficient and safe storage of substantial energy reserves.

To understand how successful adaptation to short-term high-energy intake is achieved, we turned to a unique model organism, the herbivorous fish grass carp (*Ctenopharyngodon idellus*), which exhibits low lipid requirements yet demonstrates a pronounced capacity for intraperitoneal fat accumulation and shares similarities with mammals in adipose tissue expansion patterns and pathological responses to overnutrition (20, 21, 22). Notably, grass carp exhibits exceptional capacity for rapid and safe lipid storage (23). These characteristics position grass carp as a suitable model for investigating energy storage and metabolism. We demonstrate that hypoxia inducible factor-1αa (HIF1αa)-mediated crosstalk between mature adipocytes and preadipocytes drives rapid adipocyte hyperplasia within 3 days, enabling healthy lipid storage of grass carp. In summary, our findings elucidate the physiological mechanisms underpinning grass carp's adaptation to short-term high-energy diets. This work offers novel insights into hypoxia signaling as a metabolic orchestrator in healthy fat storage and may inform strategies for addressing human obesity-related diseases stemming from excessive energy intake.

## Results

### Grass carp adapt to short-term HFD through rapid adipocyte hypertrophy and hyperplasia

**Figure 1:**
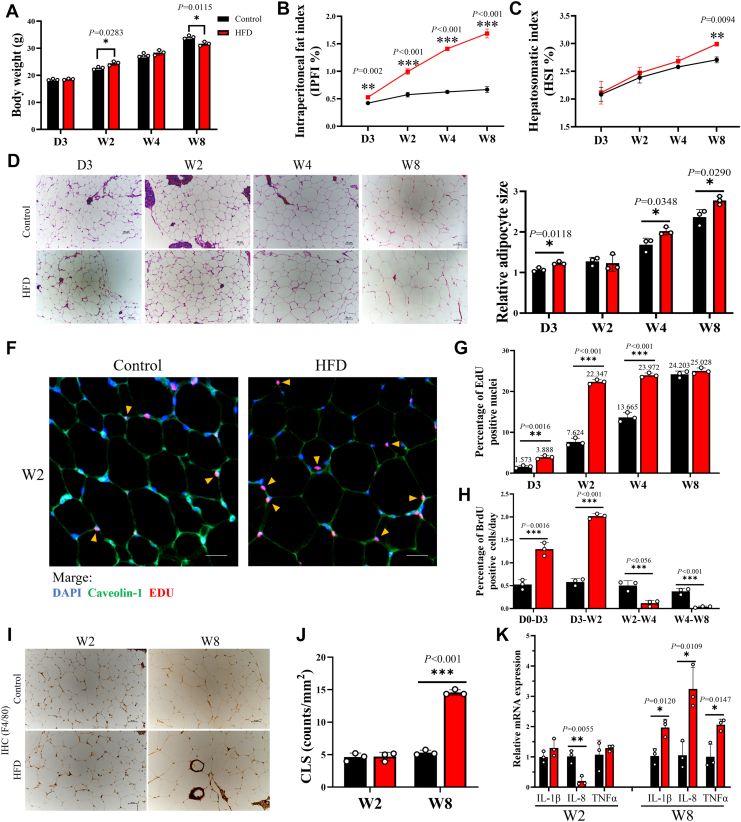
**Grass carp can adapt to short-term HFD by expanding healthy adipose tissue**. *A*, body weight from grass carp–fed Control (normal diet) and HFD (high-fat diet) for 3 days (D3), 2 weeks (W2), 4 weeks (W4), and 8 weeks (W8), n = 3. *B*, hepatosomatic from grass carp–fed Control and HFD for D3, W2, W4, and W8. Hepatosomatic index (HSI, %) = 100 × (liver weight, g)/(individual fish weight, g), n = 3. *C*, intraperitoneal fat index from grass carp–fed Control and HFD for D3, W2, W4, and W8. Intraperitoneal fat index (IPFI, %) = 100 × (intraperitoneal fat weight, g)/(final weight, g), n = 3. *D*, H&E stains of the adipose tissue from grass carp–fed Control and HFD for D3, W2, W4, and W8. Scale bar represents 50 μm. *E*, using ImageJ software to measure relative adipocyte size combined with H&E staining results of the adipose tissue, n = 3. *F*, representative images of immunofluorescence staining for EdU in the adipocyte nuclei of adipose tissue from grass carp after 1 week of EdU treatment and 2 weeks on the indicated diet. Tissue is also stained for caveolin-1 (*green*) to visualize adipocyte plasma membranes, as well as DAPI (*blue*) to visualize nuclei. Scale bar represents 50 μm. *G*, quantitative analysis of EdU positive nuclei in grass carp adipocytes after 3 days, 2 weeks, 4 weeks, and 8 weeks of conditional diet. The *yellow* arrows indicate edu-positive adipocyte nuclei, which are located within the cytoplasmic membrane of adipocytes, n = 3. *H*, growth rate of EDU-positive cell nuclei in adipocyte at different time periods, n = 3. *I*, immunohistochemical staining with an F4/80 antibody of adipose tissue, crown-like structures (CLS); scale bar represents 50 μm. *J*, quantification of CLS, n = 3. *K*, the mRNA expression of IL-1β, IL-8, TNF-α in adipose tissue, n = 3. Results are presented as means ± SD. Data analysis was conducted by Student’s two-tailed *t* test. Results with *p* < 0.05 represents a statistically significant difference: ∗*p* < 0.05; ∗∗*p* < 0.01; ∗∗∗*p* < 0.001.

To examine the tolerance limit of grass carp to high-fat diets (HFD), we performed a time-course observation in grass carp–fed a HFD. We found that short-term HFD feeding significantly enhanced growth performance in grass carp, whereas prolonged feeding (W8-HFD) resulted in growth inhibition (Fig. 1*A*). In addition, the intraperitoneal fat index began to increase by 3 days (D3) in HFD grass carp (Fig. 1*B*), but neither the HSI increased until 8 weeks in HFD grass carp (Fig. 1*C*), and liver H&E staining also showed that HFD induced liver lipid accumulation after 8 weeks (W8) (Fig. S1*A*). This suggests adipose tissue is the primary energy storage site under HFD in grass carp, and lipid ectopic deposition occurs when the adipose tissue storage capacity reaches its limit.

To further clarify the expansion pattern of adipose tissue under HFD, we stained adipose tissue with H&E and counted the area and distribution of adipocytes. Results showed that the adipocyte area increased significantly within 3 days of HFD feeding, indicating that early energy storage was accomplished primarily through adipocyte hypertrophy (Fig. 1, *D* and *E*; Fig. S1*B*). By week 2, a population of larger adipocytes emerged, accompanied by a notable increase in newly formed small adipocytes (Fig. S1*C*). This surge in small adipocytes kept the mean adipocyte area comparable to that of controls at this time point (Fig. 1*E*). At week 4, HFD induced a pronounced rightward shift in adipocyte size distribution, reflecting a higher frequency of large adipocytes (Fig. S1*D*). By week 8, the proportion of hypertrophic adipocytes further increased, while the fraction of newly formed small adipocytes markedly declined (Fig. S1*E*). Consequently, the mean adipocyte area was significantly greater in HFD-fed fish at both weeks 4 and 8 compared with controls (Fig. 1*E*). Meanwhile, the results of 5 ethynyl 2′ deoxyuridine (EdU) immunofluorescence staining showed that HFD significantly promoted the *de novo* generation of adipocytes at 3 days and 2 weeks (Fig. 1, *F*–*H*). However, after 4 and 8 weeks of HFD feeding, the number of newly generated adipocytes progressively declined, and the growth rate of EdU-positive nuclei was significantly inhibited (Fig. 1, *F*–*H*). Furthermore, under long-term HFD conditions, the decline in adipocyte hyperplasia was accompanied by increased macrophage infiltration and inflammation in adipose tissue (Fig. 1, *I*–*K*). Overall, grass carp adapt to short-term HFD by rapidly employing both adipocyte hypertrophy and hyperplasia to store excess energy. At this stage, the rapid adipocyte hyperplasia effectively prevents excessive adipocyte hypertrophy. This unique physiological characteristic underlies the capacity of grass carp to tolerate short-term high-fat diet.

### Short-term HFD activated hypoxia signaling pathway in the adipose tissue of grass carp to drive adipose tissue expansion

**Figure 2:**
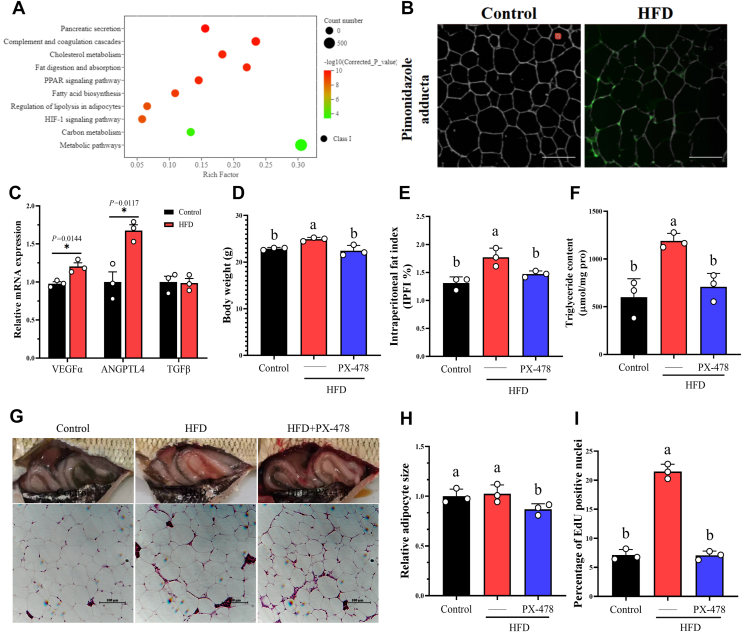
**Short-term (W2) HFD activates the HIF-1 signaling pathway in grass carp adipose tissue and promotes adipose tissue expansion**. *A*, KEGG pathway enrichment bubble plot of the top 10 differentially expressed genes identified by transcriptomic analysis of adipose tissue from grass carp after 2 weeks of Control or HFD feeding, n = 3. *B*, whole-mount immunohistochemical analysis of adipose tissue of grass carp. *Green*, pimonidazole adduct; *off-white*, adipocyte membrane; scale bar represents 100 μm *C*, the mRNA expression of HIF1αa downstream target genes VEGFα, ANGPTL4, TGFβ in the adipose tissue of grass carp after 2 weeks of Control and HFD feeding, n = 3. *D*, body weight of grass carp fed with PX-478 added to HFD for 2 weeks, n = 3. *E*, intraperitoneal fat index of grass carp fed with PX-478 added to HFD for 2 weeks, n = 3. *F*, triglyceride content in adipose tissue of grass carp fed with PX-478 added to HFD for 2 weeks, n = 3. *G*, anatomical diagram of abdominal adipose tissue of grass carp, and H&E staining of the adipose tissue from grass carp; scale bar represents 100 μm. *H*, relative adipocyte size in the adipose tissue of grass carp fed with PX-478 added to HFD for 2 weeks, n = 3. *I*, quantitative analysis of EDU-positive cell nuclei in the adipose tissue of grass carp fed with PX-478 added to HFD for 2 weeks, n = 3. Data are represented as the mean ± SD. For comparisons between two groups, a two-tailed Student’s *t* test was performed, ∗*p* < 0.05. Statistical testing was performed with one-way ANOVA for comparisons among three or more groups. The significance of the differences was assessed with *post hoc* Tukey test. Set at *p* < 0.05 shows the significant level. Different lowercase letter means significant difference among groups.

To understand the molecular mechanism underlying the adaptive expansion of adipose tissue in grass carp under short-term HFD, we performed transcriptome sequencing of adipose tissue. Interestingly, the HIF-1 signaling pathway, which is widely regarded as a hallmark of pathological conditions, was significantly enriched during this adaptive phase (Fig. 2*A*). Given its established role in regulating metabolism and cell differentiation, we focused subsequent investigations on this pathway. We used pimonidazole to stain hypoxic adducts and found that 2 weeks of HFD induced hypoxia in grass carp adipose tissue (Fig. 2*B*). Consistent with HIF1α activation, short-term HFD markedly upregulated its downstream targets VEGFα and ANGPTL4, without inducing inflammatory (IL-1β, TNFα) or fibrotic (TGFβ) genes (Figs. 1*K*, 2*C*). In contrast, long-term HFD led to concurrent upregulation of these HIF1α target genes (Figs. 1*K*, S2*A*). In addition, we also found that inhibiting the hypoxic signaling pathway in adipose tissue reversed the promoting effect of short-term HFD on grass carp growth (Fig. 2*D*) and restored the intraperitoneal fat index and adipose tissue triglyceride (TG) content to the control level (Fig. 2, *E* and *F*). To clarify whether HIF1α activated by short-term HFD affects the expansion of grass carp adipose tissue, we conducted statistical analysis on H&E staining of adipose tissue and found that inhibiting the hypoxic signaling pathway not only suppressed the increase in adipocyte size, reduced the relative number of adipocytes, but also inhibited new adipogenesis (Fig. 2, *G*–*I*). These results indicate that the hypoxic signaling pathway in grass carp adipose tissue is activated and involved in the adipocyte hyperplasia and adipocyte hypertrophy processes during short-term HFD.

### HIF1αa participated in adipocyte hypertrophy by inducing ATGL ubiquitination and degradation under short-term HFD

**Figure 3:**
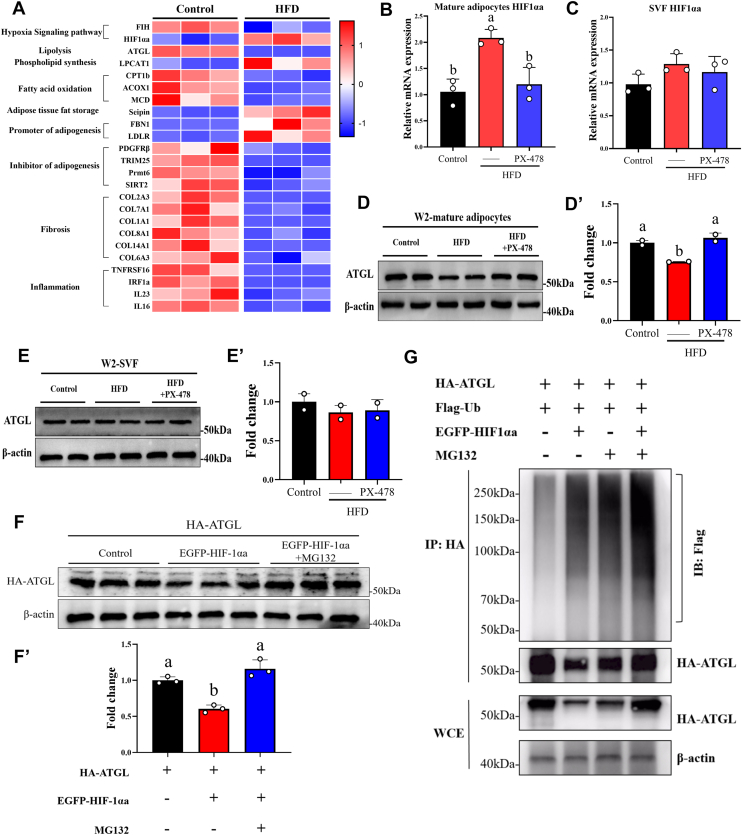
**Short-term (W2) HFD activates HIF1αa-induced ATGL ubiquitination and degradation in mature adipocyte**. *A*, heatmaps of the expression of metabolism and health-related proteins in grass carp adipose tissue following a 2-weeks feeding period with control or high-fat diet, n = 3. *B*, the mRNA expression of HIF1αa gene in freshly isolated primary mature adipocytes obtained directly from grass carp adipose tissue after 2 weeks of Control, HFD, and HFD + PX-478, n = 3. Each sample is composed of 20 fish collected from the same experimental group and tank. *C*, the mRNA expression of HIF1αa gene in freshly isolated SVF obtained directly from grass carp adipose tissue after 2 weeks of Control, HFD, and HFD + PX-478, n = 3. Each sample is composed of 20 fish collected from the same experimental group and tank. *D*, Western blot analysis of ATGL protein levels in isolated primary mature adipocytes obtained directly from grass carp adipose tissue after 2 weeks of Control, HFD, and HFD + PX-478. Each sample is composed of 20 fish collected from the same experimental group and tank. β-actin protein was used as a loading control. *D*′, quantitative analysis of immunoblots was performed, n = 2 independent experiments. *E*, Western blot analysis of ATGL protein levels in isolated SVF obtained directly from grass carp adipose tissue after 2 weeks of Control, HFD, and HFD + PX-478. Each sample is composed of 20 fish collected from the same experimental group and tank. β-actin protein was used as a loading control. *E*′, quantitative analysis of immunoblots was performed, n = 2 independent experiments. *F*, Western blot analysis of the effect of HIF1αa on the stability of ATGL. HA-ATGL and EGFP-HIF1αa plasmids were cotransfected into 293T cells and treated with MG132 (10 μM) 6 h before termination. *F*′, quantitative analysis of immunoblots was performed, n = 3. *G*, Western blot analysis of the effect of HIF1αa on the ubiquitination of ATGL. 293T cells were transfected with the indicated plasmids for 24 h, for the last 6 h in the presence of MG132 (10 μM). Data are represented as the mean ± SD. Statistical testing was performed with one-way ANOVA. The significance of the differences was assessed with *post hoc* Tukey test. Set at *p* < 0.05 shows the significant level. Different lowercase letter means significant difference among groups.

**Figure 4:**
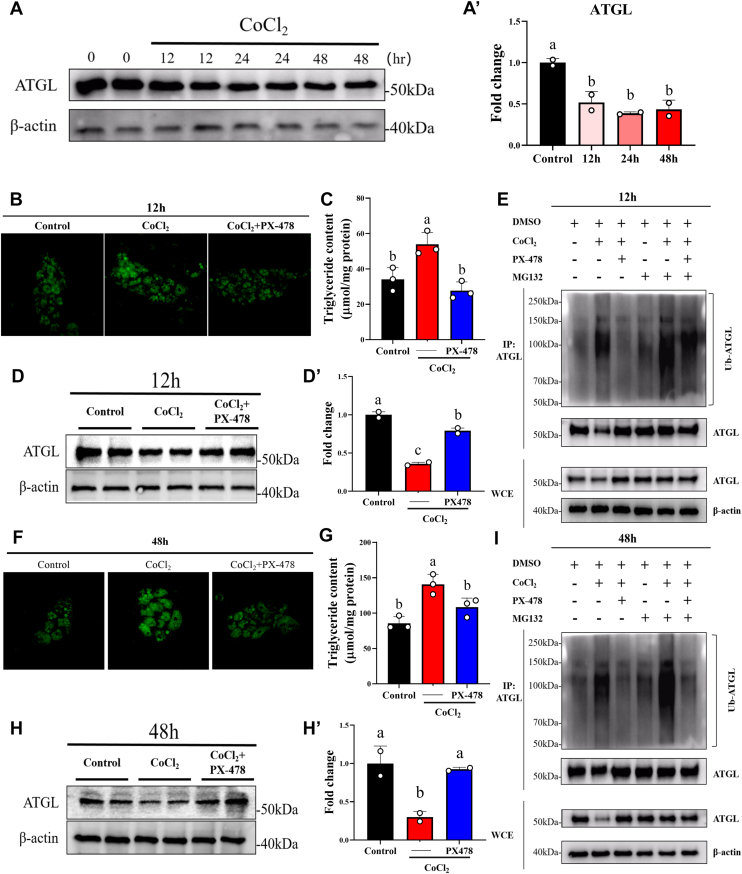
**HIF1αa promotes adipocyte hypertrophy of grass carp by degrading ATGL through the ubiquitinated proteasome pathway**. *A*, treat differentiated adipocytes (day 8) with 350 μM CoCl_2_ for 12, 24, and 48 h and detect their ATGL protein expression levels. β-actin protein was used as a loading control. *A*′, quantitative analysis of immunoblots was performed, n = 2 independent experiments. *B*, treat differentiated adipocytes with CoCl_2_ (350 μM) and PX-478 (10 μM) for 12 h and perform BODIPY staining. *C*, treat differentiated adipocytes with CoCl_2_ (350 μM) and PX-478 (10 μM) for 12 h and perform triglyceride content detection, n = 3. *D*, treat differentiated adipocytes with CoCl_2_ (350 μM) and PX-478 (10 μM) for 12 h and detect their ATGL protein expression levels. β-actin protein was used as a loading control. *D*′, quantitative analysis of immunoblots was performed, n = 2 independent experiments. *E*, treat differentiated adipocytes with CoCl_2_ (350 μM) and PX-478 (10 μM) for 12 h and detect the ubiquitination level of ATGL by IP. MG132 (10 μM) was added 6 h before sample collection. *F*, treat differentiated adipocytes with CoCl_2_ (350 μM) and PX-478 (10 μM) for 48 h and perform BODIPY staining. *G*, treat differentiated adipocytes with CoCl_2_ (350 μM) and PX-478 (10 μM) for 48 h and perform triglyceride content detection, n = 3. *H*, treat differentiated adipocytes with CoCl_2_ (350 μM) and PX-478 (10 μM) for 48 h and detect their ATGL protein expression levels. β-actin protein was used as a loading control. *H*′, quantitative analysis of immunoblots was performed, n = =2 independent experiments. *I*, treat differentiated adipocytes with CoCl_2_ (350 μM) and PX-478 (10 μM) for 48 h and detect the ubiquitination level of ATGL by IP. MG132 (10 μM) was added 6 h before sample collection. Data are represented as the mean ± SD. Statistical testing was performed with one-way ANOVA. The significance of the differences was assessed with *post hoc* Tukey test. Set at *p* < 0.05 shows the significant level. Different lowercase letter means significant difference among groups.

To further elucidate how short-term HFD induces adipocyte hypertrophy following the activation of hypoxic signaling in grass carp adipose tissue, we performed proteomic profiling of the adipose tissue (Table S1). The results showed that HIF-1αa was significantly upregulated and adipose triglyceride lipase (ATGL) was significantly downregulated after short-term HFD feeding (Fig. 3*A*). As the primary rate-limiting enzyme initiating intracellular TG hydrolysis, decreased ATGL leads to lipid droplet accumulation (24, 25). We therefore hypothesized that the upregulation of HIF-1αa mediates adipocyte hypertrophy under short-term HFD by leading to the downregulation of ATGL. In addition, proteins related to fat storage were significantly upregulated (Fig. 3*A*). However, short-term HFD–induced adipocyte hypertrophy did not cause inflammation and fibrosis of adipose tissue (Fig. 3*A*). This indicates that short-term HFD–induced adipocyte hypertrophy is adaptive adipocytes enlargement. Notably, we isolated mature adipocytes and stromal vascular fraction (SVF) in grass carp adipose tissue and found that the activation of HIF1αa by short-term HFD occurs specifically in mature adipocytes and not in SVF (Fig. 3, *B* and *C*). The decrease in ATGL protein also occurs specifically in mature adipocytes (Fig. 3, *D* and *E’*). Therefore, we speculate that HIF1αa in mature adipocytes may participate in the degradation of ATGL protein after HFD activation. To uncover how changes in HIF1αa levels led to decrease in ATGL levels, we focused on ubiquitination and proteasome-targeted degradation. We used HEK293T cells to co-expressing EGFP-HIF1αa and HA-ATGL. Western blot results shown that HIF1αa induced a decrease in ATGL protein levels, which returned to control levels following the addition of the proteasome-specific inhibitor MG132 (Fig. 3, *F* and *F’*). This means that HIF1αa induces the degradation of ATGL and is regulated by the proteasome-mediated degradation pathway. Subsequently, we used a ubiquitination assay to test whether ATGL was ubiquitinated and then degraded through the proteasome pathway. As shown in Figure 3*G*, when EGFP-HIF1αa, HA-ATGL, and Fiag-Ub are co-expressed, the poly-ubiquitination of ATGL was increased both in the presence or absence of MG132, which indicates that HIF1αa induces polyubiquitination of ATGL. Furthermore, ATGL ubiquitination levels further increased after adding MG132 indicates that ATGL is degraded through the ubiquitination proteasome pathway (Fig. 3*G*). To clarify whether the activation of HIF1αa in mature adipocytes of grass carp induces ATGL protein degradation through the ubiquitination proteasome pathway, leading to adipocyte hypertrophy, we treated differentiated adipocytes with CoCl_2_ (HIF-1αa) and found that the protein level of ATGL decreased at 12 h and persisted for 48 h (Fig. 4, *A* and A′). BODIPY staining and TG results showed that PX-478 restored CoCl_2_-induced lipid droplet enlargement (Fig. 4, *B* and *C*) and TG accumulation in differentiated adipocytes (Fig. 4, *F* and *G*). In addition, the reduction of ATGL protein induced by CoCl_2_ was also restored to the control level after adding HIF1αa inhibitor (Fig. 4, *D*–*H’*). We further found that the decrease in ATGL protein in grass carp adipocytes is due to degradation through the ubiquitination proteasome pathway. CoCl_2_ induced ubiquitination of ATGL in differentiated adipocytes of grass carp and was improved after the addition of PX-478. Meanwhile, the ubiquitination level of ATGL was further enhanced after adding MG132 (Fig. 4, *E*–*I*). Taken together, our data demonstrate that short-term HFD drives differentiated adipocytes hypertrophy by activating HIF1αa in differentiated adipocytes and inducing ATGL ubiquitination and degradation.

### HIF1αa promoted transcription of ATGL to maintain basal lipolysis in mature adipocyte under short-term HFD

**Figure 5:**
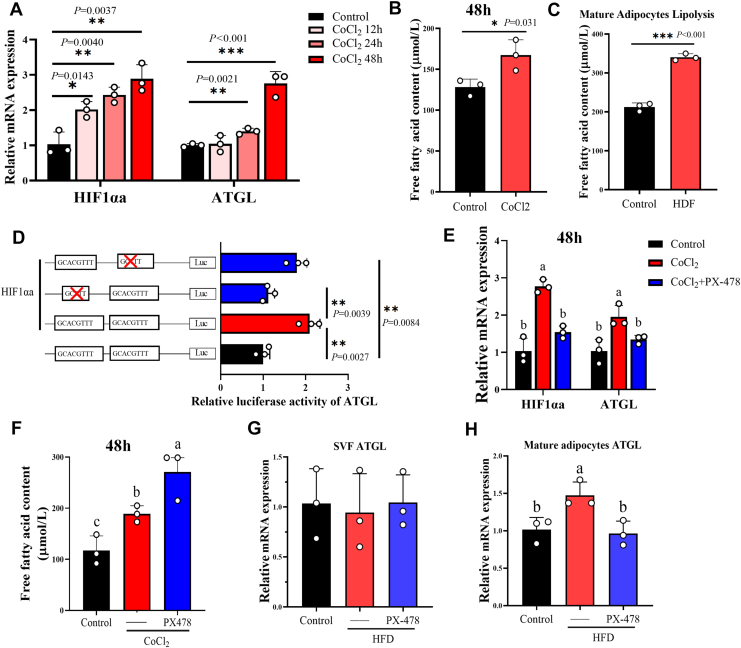
**HIF1αa promotes ATGL mRNA expression through transcriptional regulation, maintaining basal lipolysis in mature adipocyte**. *A*, treat differentiated adipocytes with 350 μM CoCl_2_ for 12, 24, and 48 h and detect the expression levels of HIF1αa and ATGL mRNA in the cells, n = 3. *B*, treat differentiated adipocytes with 350 μM CoCl_2_ for 48 h and collect the culture medium to detect free fatty acid (FFA) content, n = 3. *C*, *ex vivo* FFA release by mature adipocytes isolated from HFD-fed grass carp for 2 weeks, n = 3. *D*, site-mutation analysis of ATGL-binding sites on pGL3.0-HIF1αa vectors in 293 T cells, n = 3. *E*, treat differentiated adipocytes with 350 μM CoCl_2_ and PX-478 (10 μM) for 48 h and detect the expression levels of HIF1αa and ATGL mRNA in the cells, n = 3. *F*, treat differentiated adipocytes with CoCl_2_ (350 μM) and PX-478 (10 μM) for 48 h and collect the culture medium to detect free fatty acid (FFA) content, n = 3. *G*, the mRNA expression of ATGL gene in freshly isolated SVF obtained directly from grass carp adipose tissue after 2 weeks of Control, HFD, and HFD + PX-478, n = 3. Each sample is composed of 20 fish collected from the same experimental group and tank. *H*, the mRNA expression of ATGL gene in freshly isolated primary mature adipocytes obtained directly from grass carp adipose tissue after 2 weeks of Control, HFD, and HFD + PX-478, n = 3. Each sample is composed of 20 fish collected from the same experimental group and tank. Data are represented as the mean ± SD. For comparisons between two groups, a two-tailed Student’s *t* test was performed, ∗*p* < 0.05; ∗∗*p* < 0.01; ∗∗∗*p* < 0.001. Statistical testing was performed with one-way ANOVA for comparisons among three or more groups. The significance of the differences was assessed with *post hoc* Tukey test. Set at *p* < 0.05 shows the significant level. Different lowercase letter means significant difference among groups.

Interestingly, as shown in Figure 4*A*, the ATGL protein level in differentiated adipocytes did not decrease further with CoCl_2_ treatment time, but stabilized at a relatively low level. We propose that compensatory transcriptional activation of ATGL maintains baseline ATGL protein levels, preventing excessive adipocyte hypertrophy. Thus, we detected the expression levels of ATGL mRNA in differentiated adipocytes treated with CoCl_2_ for 12 h, 24 h, and 48 h. The results indicate that ATGL mRNA expression in differentiated adipocytes was significantly upregulated at 24 h and 48 h treated with CoCl_2_ treatment (Fig. 5*A*). Meanwhile, CoCl_2_ treatment significantly increased the release of free fatty acids (FFAs) from differentiated adipocytes (Fig. 5*B*). It is worth noting that following a 4-h culture of mature adipocytes isolated from short-term HFD-fed grass carp, we detected the FFA content in the medium. The results showed a significant increase in FFA release from the HFD group compared with the control (Fig. 5*C*). We also found through dual-luciferase reporter assay that HIF1αa increased ATGL promoter activity, and ATGL promoter activity decreased after mutation of the HIF1αa-binding site (Fig. 5*D*). This further confirms the activating effect of HIF1αa on ATGL transcription. In addition, we found that the level of ATGL mRNA was also inhibited after HIF1αa inhibition, but the release of FFA from lipolysis was further increased (Fig. 5, *E* and *F*). This imbalance reflects HIF1αa′s dominant effect on protein degradation over transcriptional promotion. It is worth mentioning that the promoting effect of short-term HFD on ATGL mRNA expression also occurs only in mature adipocytes rather than SVF (Fig. 5, *G* and *H*).

Taken together, HIF1αa in grass carp adipose tissue was activated under HFD conditions. On one hand, HIF1αa induced the degradation of ATGL protein. On the other hand, HIF1αa stimulated ATGL mRNA expression to maintain ATGL protein levels, thereby supporting the body's essential lipolysis while preventing excessive adipocyte hypertrophy.

### PPARγ-mediated preadipocyte differentiation was activated by FFA released from mature adipocyte

**Figure 6:**
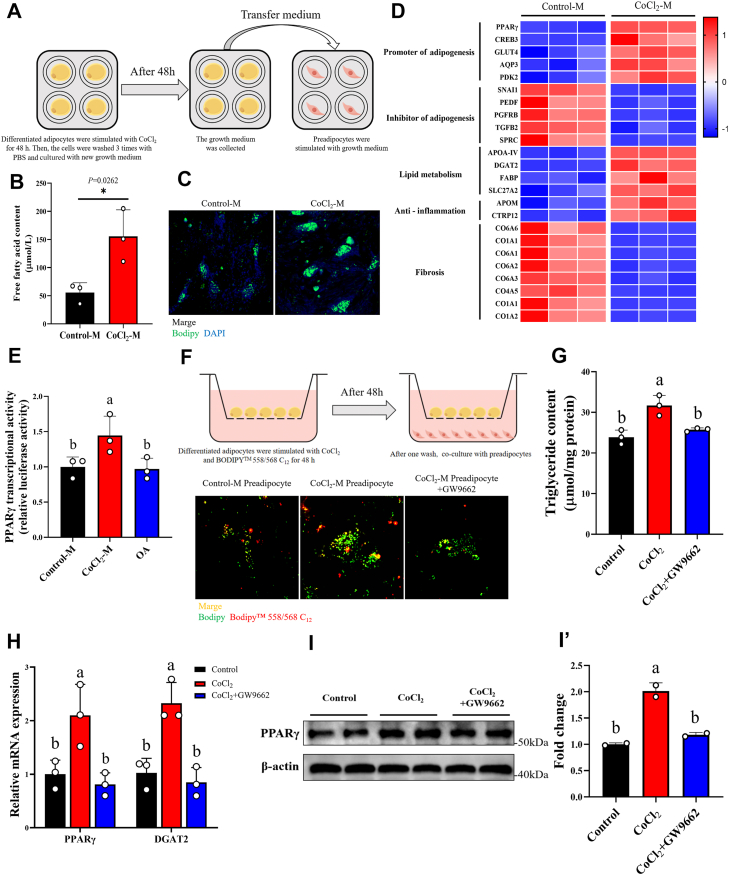
**HIF1αa induces the release of FFA from mature adipocytes through basal lipolysis, which activates PPARγ in preadipocytes, promoting adipocyte hyperplasia**. *A*, schematic drawing of the experimental procedures. *B*, treat differentiated adipocytes with 350 μM CoCl_2_ for 48 h; the growth medium was replaced to continue culturing the mature adipocytes for an additional 48 h, and the medium was collected to measure the free fatty acid (FFA) content, n = 3. *C*, BODIPY staining of grass carp primary preadipocytes under different medium treatment conditions, Bodipy (*green*) and nuclei (*blue*). *D*, differential genes related to adipogenesis, lipid metabolism, inflammation, and fibrosis in transcriptome sequencing of preadipocytes under different culture media treatment conditions, n = 3. *E*, transcriptional activity of PPAR γ under different culture media and OA treatment conditions, n = 3. *F*, the differentiated adipocytes were treated with 350 μM CoCl_2_ and 1 μM BODIPY 558/568 C_12_ (a fluorescent fatty acid tracer) (*red*) for 48 h to induce lipolysis and FFA labeling. After washing to remove residual compounds, differentiated adipocytes were cocultured with preadipocytes for 48 h. Use Bodipy (*green*) staining and take photos with a confocal laser microscope. Control group: preadipocytes cocultured with differentiated adipocytes that were not treated with CoCl_2_; CoCl_2_ group: preadipocytes cocultured with differentiated adipocytes that were treated with CoCl_2_; CoCl_2_ + GW9662 group: preadipocytes cocultured with differentiated adipocytes that were treated with CoCl_2_ and then treated with GW9662. *G*, TG content of preadipocytes under different coculture systems and composite treatment conditions, n = 3. *H*, the mRNA expression levels of PPARγ and DGAT2 in preadipocytes under different coculture systems and composite treatment conditions, n = 3. *I*, Western blot of PPARγ in preadipocytes under different coculture systems and composite treatment conditions. β-actin protein was used as a loading control. *I*′, quantitative analysis of immunoblots was performed, n = 2 independent experiments. Data are represented as the mean ± SD. For comparisons between two groups, a two-tailed Student’s *t* test was performed, ∗*p* < 0.05. Statistical testing was performed with one-way ANOVA for comparisons among three or more groups. The significance of the differences was assessed with *post hoc* Tukey test. Set at *p* < 0.05 shows the significant level. Different lowercase letter means significant difference among groups.

The short-term HFD not only promoted the hypertrophy of grass carp adipocytes but also promoted adipocyte hyperplasia. Previous studies have shown that FFA released by lipolysis can be re-esterified and form lipid droplets (26). Consequently, we speculate that adipocyte hyperplasia (preadipocyte differentiation) may be activated by FFA secreted from lipolysis during mature adipocytes hypertrophy under short-term HFD. Therefore, we collected differentiated adipocytes culture medium according to Figure 6*A* to incubate preadipocytes. We found that the FFA in the differentiated adipocytes culture medium after replacement significantly increased following CoCl_2_ treatment and promoted preadipocyte differentiation (Fig. 6, *B* and *C*). The transcriptome results also showed that genes related to promoting adipogenesis were significantly upregulated in preadipocytes, including PPARγ (the main transcriptional regulator of adipocyte differentiation) (Fig. 6*D*). To better understand that PPARγ was activated by FFA released during differentiated adipocytes hypertrophy, we used dual-luciferase assay to verify. The results indicated that media from CoCl_2_-induced hypertrophic adipocytes significantly activated PPARγ transcriptional activity compared to media from normal adipocytes (Fig. 6*E*). In addition, we used cell co-culture and BODIPY-C12 to track FFA secreted by differentiated adipocytes (Fig. 6*F*). As shown in Figure 4*E*, FFA released by differentiated adipocytes can be taken up by preadipocytes, and CoCl_2_-induced hypertrophic adipocytes significantly promoted the differentiation of preadipocytes (Fig. 6*F*). We further performed chromatin immunoprecipitation sequencing for PPARγ in preadipocytes cocultured with differentiated adipocytes (treated with or without CoCl_2_) (Fig. S3*A*, Table S2). Differential peak analysis identified 1744 PPARγ-binding sites that were specifically enriched in the CoCl_2_ treatment group and located within 4.5 kb of the predicted transcription start sites (Fig. S3*B*). Notably, PPARγ occupancy was detected at genomic regions of known adipogenic target genes, including 3- hydroxy-3-methylglutaryl-Coenzyme A synthase 1 (HMGCS1) and fat storage inducing transmembrane protein 1 (FITM1) (Fig. S3*C*). This indicates that PPARγ exhibits higher chromatin association in preadipocytes cocultured with CoCl_2_-treated differentiated adipocytes, which may drive the expression of its target genes more strongly compared with Control group. Meanwhile, the TG and differentiation-related genes of preadipocytes showed that CoCl_2_-treated hypertrophic adipocytes significantly enhanced preadipocyte differentiation and TG accumulation, but these effects were restored to control levels upon the addition of GW9662 (Fig. 6, *G* and *H*). The protein expression of PPARγ was also promoted by CoCl_2_-treated hypertrophic adipocytes and back to normal after GW992 treatment (Fig. 6, *I* and *I’*). Notably, the transcriptional activity of PPARγ is not affected by oleic acid (Fig. 6*E*). These results demonstrate that PPARγ in preadipocytes is activated by FFA released during differentiated adipocytes hypertrophy rather than exogenous fatty acids in the diet. It is precisely because the timely differentiation of preadipocytes avoids excessive hypertrophy of differentiated adipocytes and increases the energy storage limit of adipose tissue.

### Inhibition of PPARγ-mediated adipocyte hyperplasia disrupts grass carp's adaptability to short-term HFD

**Figure 7:**
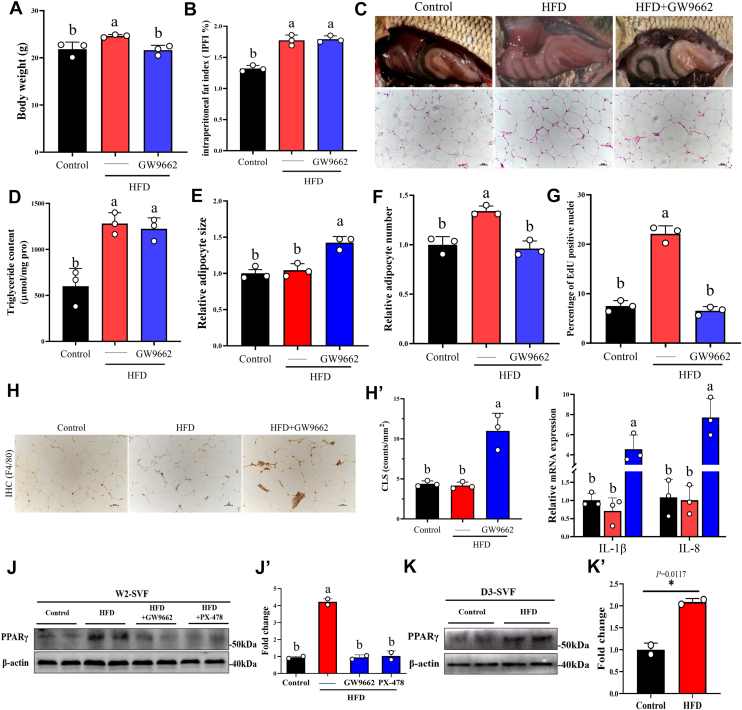
**Inhibition of adipocyte hyperplasia and induction of pathological expansion of adipose tissue under short-term HFD conditions**. *A*, body weight of grass carp fed with GW9662 added to HFD for 2 weeks, n = 3. *B*, intraperitoneal fat index of grass carp fed with GW9662 added to HFD for 2 weeks, n = 3. *C*, anatomical diagram of abdominal adipose tissue of grass carp and its H&E staining; scale bar represents 50 μm. *D*, triglyceride content in the adipose tissue of grass carp fed with GW9662 added to HFD for 2 weeks, n = 3. *E*, relative adipocyte size in the adipose tissue of grass carp fed with GW9662 added to HFD for 2 weeks, n = 3. *F*, relative adipocyte number in the adipose tissue of grass carp fed with GW9662 added to HFD for 2 weeks, n = 3. *G*, quantitative analysis of EDU-positive cell nuclei in the adipose tissue of grass carp fed with GW9662 added to HFD for 2 weeks, n = 3. *H*, immunohistochemical staining with an F4/80 antibody of adipose tissue, crown-like structures (CLS); scale bar represents 50 μm. *H*′, quantification of CLS, n = 3. *I*, the mRNA expression of IL-1β, IL-8 in adipose tissue, n = 3. *J*, Western blot analysis of PPARγ protein levels in isolated SVF obtained directly from grass carp adipose tissue after 2 weeks of Control, HFD, HFD + PX-478, and HFD + GW9662. Each sample is composed of 20 fish collected from the same experimental group and tank. β-actin protein was used as a loading control. *J*′, quantitative analysis of immunoblots was performed, n = 2 independent experiments. *K*, Western blot analysis of PPARγ protein levels in isolated SVF obtained directly from grass carp adipose tissue after 3 days of Control and HFD. Each sample is composed of 20 fish collected from the same experimental group and tank. β-actin protein was used as a loading control. *K*′, quantitative analysis of immunoblots was performed, n = 2 independent experiments. Data are represented as the mean ± SD. For comparisons between two groups, a two-tailed Student’s *t* test was performed, ∗*p* < 0.05. Statistical testing was performed with one-way ANOVA for comparisons among three or more groups. The significance of the differences was assessed with *post hoc* Tukey test. Set at *p* < 0.05 shows the significant level. Different lowercase letter means significant difference among groups.

Given the crucial role of adipocyte hyperplasia in grass carp treatment with HFD (22), we added GW9662 to short-term HFD to inhibit adipocyte hyperplasia. We found through *in vivo* studies that adding PPARγ inhibitors to short-term HFD not only inhibits the weight gain of grass carp but also induces the accumulation of intraperitoneal fat in grass carp (Fig. 7, *A*–*D*). We further analyzed the expansion pattern of adipose tissue and found that GW9662 reversed the activation effect of short-term HFD on adipocyte hyperplasia, leading to excessive adipocyte hypertrophy (Fig. 7, *E*–*G*). Importantly, inhibition of adipocyte hyperplasia *via* PPARγ inhibitors induced adipose tissue macrophage infiltration and inflammation under short-term HFD (Fig. 7*I*). Furthermore, we found that the inhibition of HIF1αa under short-term HFD conditions also reversed the enhancing effect of HFD on PPARγ protein expression in grass carp preadipocytes (Fig. 7, *J* and *J’*). It is worth mentioning that HFD has promoted the protein expression of PPARγ in grass carp preadipocytes as early as the 3 days (Fig. 7, *K* and *K’*). The above results indicate that grass carp can adapt to short-term HFD mainly due to the rapid occurrence of adipocyte hyperplasia, which increases the energy storage limit of grass carp adipose tissue and alleviates excessive hypertrophy of existing adipocytes. In brief, cross-talk between adipocyte hypertrophy and hyperplasia through HIF1αa-ATGL-PPARγ helps grass carp adapt to short-term HFD.

## Discussion

Adipose tissue, the primary energy storage organ in vertebrates, grows though adipocyte hyperplasia, adipocyte hypertrophy, or both. Similar to mammals (17), both adipocyte hyperplasia and adipocyte hypertrophy contribute to adipose tissue expansion in grass carp fed with HFD, indicating that these two processes are conserved in vertebrates. It has been hypothesized that upon attaining maximal size, adipocytes stimulate the production of new adipocytes (27, 28). However, our data and the results in mouse (17) demonstrate that adipocyte hyperplasia is initiated at the onset of diet-induced obesity, challenging this viewpoint.

Adipocyte hyperplasia and hypertrophy contribute differentially to metabolic health. Previous studies have shown that adipocyte hypertrophy resulting from impaired adipocyte hyperplasia leads to metabolic diseases in fish and mammals (13, 14, 21). However, promoting adipocyte hyperplasia alleviates the negative effects of long term-HFD in grass carp (22). These results indicate that adipocyte hyperplasia–dominated adipose tissue expansion is critical for high-energy tolerance in grass carp. Consistent with this, we observed rapid adipocyte hyperplasia specifically during the HFD-tolerant phase in grass carp. This rapid adipocyte hyperplasia response increases fat storage capacity, effectively avoids excessive hypertrophy of mature adipocytes, and their potential pathological consequences in the short term. It thus constitutes a key adaptive strategy for short-term high-energy intake tolerance in grass carp. This strategy stands in sharp contrast to the response in mammals: whereas grass carp initiate hyperplasia within 3 days of high-energy feeding, mice typically require 6 to 8 weeks for a detectable hyperplastic response under HFD conditions (17, 18, 19). Critically, mice can exhibit adipose tissue inflammation as early as 3 days post-HFD initiation (29). Hence, the delayed hyperplastic response in mammals may be a key factor contributing to their early susceptibility to HFD-induced metabolic dysfunction. Meanwhile, these findings further suggest that grass carp may represent a valuable model for investigating mechanisms underlying short-term tolerance to HFD.

Our data establish that in grass carp, adipocyte hypertrophy during short-term high-energy feeding is driven by HIF1α-mediated ubiquitination and subsequent proteasomal degradation of ATGL. This finding reveals a conserved mechanism across species, as an identical HIF1α–ATGL proteolytic axis has been reported to promote lipid storage in both mammalian adipocytes and *Caenorhabditis elegans* (30). Thus, the inhibition of ATGL protein stability by HIF1α emerges as an evolutionarily conserved molecular pathway underlying adipocyte hypertrophy in vertebrates and beyond. However, a key species-specific adaptation lies in the transcriptional regulation of ATGL. In mammalian models, hypoxia typically suppresses ATGL expression (31). In contrast, we found that HIF1αa in grass carp robustly upregulates its transcription. This distinct response may be attributed to the functional diversification of teleost HIF1α subtypes (HIF1αa and HIF1αb) following gene duplication. Thus, HIF1αa in grass carp uniquely exerts a dual regulatory mode: it simultaneously promotes ATGL protein degradation and upregulates ATGL transcription.

The maintenance of basal ATGL-mediated lipolysis, *via* this HIF1αa-driven transcriptional upregulation, creates a critical link to adipocyte hyperplasia. PPARγ is a key gene that regulates the differentiation of preadipocytes and requires FFA as its activating ligand (22, 31, 32). In mammalian systems, ATGL-mediated lipolysis has been shown to supply lipid-derived ligands necessary for PPARγ activation and TG synthesis in white adipose tissue (33, 34). Similarly, in the teleost grass carp, we found that PPARγ-driven adipogenesis also depends on ATGL-mediated lipolysis in adipocytes. This indicates that FFAs released through ATGL-dependent lipid breakdown serve as key signaling molecules coordinating crosstalk between mature adipocytes and preadipocytes. This also provides direct evidence for the hypothesis that signal(s) from hypertrophied adipocytes promote the subsequent lipid filling of activated adipocyte precursors. Notably, in human and murine systems, mature adipocytes have been reported to inhibit preadipocyte differentiation *in vitro* (35), suggesting that disruption of this inhibitory crosstalk may represent a key barrier to healthy adipose expansion under nutrient excess. Taken together, our findings position ATGL-mediated basal lipolysis as a conserved but differentially regulated node linking hypoxia signaling to adipocyte hyperplasia.

Based on the bidirectional regulation of ATGL by HIF1αa mentioned above and the high-fat tolerance phenotype of grass carp, we identified HIF1αa as essential for healthy adipose expansion in HFD-tolerant grass carp. In mammals, white adipose tissue hypoxia in obese mice leads to altered adipocyte function; this dysregulation may underlie the development of obesity-associated disorders (1, 36, 37). In contrast to mammalian models, short-term HFD–induced hypoxia in grass carp adipose tissue does not trigger inflammatory responses. Given that excessive adipocyte hypertrophy triggers inflammation (9, 21), we propose that this rapid adipocyte hyperplasia response driven by hypoxia/HIF1αa signaling disperses lipid storage load by increasing the number of adipocytes, effectively preventing excessive hypertrophy of individual adipocytes and suppressing the inflammatory response triggered by excessive adipocyte hypertrophy. This implies hypoxia signaling is not invariably pathogenic: whereas HIF1α signifies metabolic stress in mammals (38, 39), it facilitates short-term HFD adaptation in grass carp. Thus, our findings provide insight for human obesity research by reframing the role of hypoxia. While adipose tissue hypoxia in obesity is associated with inflammation and dysfunction, our findings suggest that the adverse effects may not be hypoxia itself but the impaired ability of hypoxic signals to initiate adipocyte hyperplasia. This suggests a therapeutic strategy aimed at enhancing the downstream hyperplasia response to hypoxia in human adipose tissue could promote healthier energy storage.

While this study provides mechanistic insights into hypoxia-coordinated healthy adipose expansion, we acknowledge a limitation: tissue oxygen levels were not directly measured. Instead, we relied on pimonidazole hydrochloride staining and determination of HIF-1α target genes. The future use of direct measurement of tissue oxygen levels for detection work can further support the research conclusions of this study. More importantly, our findings prompt a key mechanistic question: what regulates the decline in adipocyte hyperplasia as grass carp transition from the short-term tolerant phase to long-term HFD intolerance? Investigating whether this results from precursor pool exhaustion, the accumulation of inhibitory signals, or other regulatory changes could provide important comparative insights. Elucidating this regulatory switch may thus provide a valuable comparative model for understanding the common challenge of restricted adipocyte hyperplasia in human obesity.

In summary, this study found that short-term high-energy diets activation triggers HIF1αa signaling in the mature adipocyte of grass carp. This pathway not only downregulates ATGL protein *via* the ubiquitin-proteasome system to induces adipocyte hypertrophy but also maintains baseline lipolysis by upregulating ATGL transcription, thus activating PPARγ-driven adipocyte hyperplasia and distributing storage burden. Our findings in grass carp reveal an alternative strategy for healthy adipose expansion characterized by rapid hypertrophy hyperplasia coordination, potentially evolved to cope with episodic high-energy availability. This contrasts with the delayed and often dysregulated expansion observed in mammals under chronic overnutrition, highlighting the importance of the timing and coordination of hyperplastic and hypertrophic responses in determining metabolic health outcomes across vertebrates.

## Experimental procedures

### Animal studies

Grass carp were obtained from Ankang Fisheries Experimental Demonstration Station. Grass carp kept in pond cages with a temperature range of 23 to 28 °C, pH at 7.5 to 8.0, dissolved oxygen levels between 7 to 11 mg/L, and ammonia nitrogen concentrations below 0.05 mg/L. All animal procedures were approved by the Animal Management Committee of Northwest A&F University (NWAFU-DK-20220606). All experiments performed with weight-matched grass carp (19.05 ± 0.06 g). The grass carp were fed regularly and quantitatively at 08:00, 12:00, and 16:00 each day for 8 weeks. Five experimental diets were prepared in this experiment: Control group (protein 33%, lipid 4%), HFD group (protein 33%, lipid 8%), HFD + PX-478 (HY-10231, MCE) group (protein 33%, lipid 8% + PX-478 60 mg/kg), and HFD + GW9662 (HY-16578, MCE) group (protein 33%, lipid 8% + GW9662 60 mg/kg). Each experimental group is set up with three repeated fish tanks, each containing 30 grass carp. Ultimately, each fish tank will be treated as an independent biological sample. In addition, according to the experimental requirements, samples were collected for testing on the third day (D3), second week (W2), fourth week (W4), and eighth week (W8) after the start of feeding.

### Grass carp primary adipocytes isolation and treatment

The isolation and culture methods of grass carp primary adipocytes were as described previously (22). The isolated preadipocytes were cultured in growth medium for 7 days until reaching confluence, then induced to differentiate using differentiation medium for 8 days until the terminal differentiation stage. Different adipocytes cultured to terminal differentiation were treated with CoCl_2_ (350 μM), PX-478 (10 μM), and GW9662 (10 μM) for 48 h. It is worth noting that MG132 (10 μM, HY-13259, MCE) should be added after 36 h when detecting ubiquitination.

### Grass carp mature adipocytes and SVF preparation and culture

Briefly, after 2 weeks of feeding, experimental fish were anesthetized, and mesenteric adipose tissue was immediately collected under sterile conditions and transferred into culture medium. Each sample is composed of 20 fish collected from the same experimental group and tank. The tissue was carefully washed twice with PBS, minced into small fragments, and subsequently digested with type I collagenase for 30 min. Following digestion, the cell suspension was passed through a 200-μm nylon mesh to remove undigested debris. The filtrate was then centrifuged at 700×*g* for 10 min. Mature adipocytes floating at the top of the tube were gently aspirated and transferred to fresh tubes, followed by three rounds of washing by flotation before further use. In parallel, the pellet obtained after centrifugation, corresponding to the SVF, was washed three times and subsequently used for experiments.

### Basal lipolysis analysis of grass carp mature adipocytes

Mature adipocytes were obtained from the mesenteric adipose tissue of grass carp and suspended in Krebs–Hepes buffer supplemented with 2% bovine serum albumin. The cells were incubated in polypropylene tubes at a final density of 6 × 10ˆ5 cells/ml in a total volume of 1 ml. Incubations were carried out at 28 °C under an atmosphere of 5% CO_2_ for 4 h, with three independent tubes prepared for each time point. Upon completion of the incubation, the tubes were immediately transferred to ice for 2 min to terminate cellular activity. The samples were then centrifuged briefly at 700×*g* for 5 min at 4 °C, after which the supernatant was carefully collected. Concentrations of glycerol and FFAs in the cell-free medium, serving as indicators of lipolytic activity, were determined using an ultrasensitive commercial assay kit (BC0594, Solarbio).

### HEK293T culture transfection and treatment

HEK293T (ab255449, Abcam) cells were cultured in Dulbecco's modified eagle medium high-glucose medium containing 10% fetal bovine serum and placed in an incubator with a temperature of 37 °C and 5% CO_2_. Until the cell density reached 70%, according to the manufacturer's instructions, plasmid transfection was performed using Lipo8000 (C0533, Beyotime) liposome transfection reagent at the required concentration and HEK293T cells were cultured for 48 h. MG132 was added 6 h before sample collection.

### H&E staining and immunohistochemistry

The adipose tissue samples were dissected and preserved in a solution of 4% paraformaldehyde. After being fixed at 4 degrees for 24 h, the tissue samples were handed over to Chengdu Lilai Biotechnology Co., Ltd for H&E staining. Finally, the stained tissue sections were visualized and captured using an upright microscope manufactured by Leica Biosystems in Germany, and the adipocyte area within these tissue sections was measured using ImageJ software.

For immunofluorescence staining, adipose tissue paraffin sections were blocked with bovine serum albumin and incubated overnight with F4/80 antibody (1:200, bs-11182R, Bioss) at 4 °C to specifically bind to macrophages. After washing three times with PBS, incubate with the corresponding second antibody at room temperature for 1.5 h. Finally, immunohistochemical sections were imaged on an upright microscope and the macrophage clusters of the crown-like structures were quantitatively analyzed using ImageJ software.

### EDU assay

Follow the instructions of the EDU-488 Cell Proliferation Detection Kit for operation (C0075L, Beyotime). In short, fed the experimental fish with EDU (4 mg/kg added to the diet) and continue for 1 week before feeding the experimental diets. Adipose tissue was collected on the third day, second week, fourth week, and eighth week after feeding the experimental diet, washed with PBS to remove blood and residual EDU, and paraffin sections were prepared using standard procedures. Subsequently, the sections were eluted with xylene and an ethanol gradient, as well as PBS. The Click reaction solution was then applied and incubated at room temperature for 30 min in the dark before washing with PBS. Antibody staining for the membrane protein Caveolin-1 (1:400, 16447-1-AP, Bioss) was performed, followed by DNA staining with DAPI solution. Finally, the sections were observed and images were captured using a Laser Scanning Confocal Microscopy. Adipocyte nuclei are indicated with arrowheads (yellow indicates EDU-positive) and are identified by their location inside the adipocyte plasma membrane (green indicates Caveolin-1–labeled adipocyte membrane).

### Fatty acid–conjugated BODIPY tracer experiments

First, mature adipocytes were incubated with Bodipy 558/568 C12 (red) (1 μM, D3835, Thermo Fisher Scientific) for 48 h to fluorescently label preformed lipid droplets. After 48 h, mature adipocytes were washed one times with PBS. Subsequently, fluorescently labeled mature adipocytes were cocultured with preadipocytes for 48 h after washing the cells 1 to 2 times with PBS and stain lipid droplets in preadipocytes with 2 μM BODIPY (green) (C2053S, Beyotime). Finally, green fluorescence and red fluorescence were observed under excitation light at 488 nm and 568 nm using a confocal laser microscope (Leica) to clarify the entry of FFA released by mature adipocytes into preadipocytes and their involvement in lipid droplets formation.

### *In vivo* detection of hypoxia

One hour prior to euthanasia, fish were injected with the hypoxia marker pimonidazole hydrochloride (60 mg/kg) (HPI-100Kit, Hypoxyprobe). After 1 hour, grass carp adipose tissue was collected and fixed with 4% paraformaldehyde.

Unstained paraffin-embedded tissue samples were probed with 1:50 FITC-conjugated IgG1 mouse monoclonal antibody clone and stained with 1:150 rabbit anti-FITC HRP. All operations are carried out according to the requirements of the kit.

### TG and FFA content determination

Tissues/cells TG and FFA content was measured using the Triglyceride Detection Kit (E1013, Applygen) and Free Fatty Acids Detection Kit (BC0594, Solarbio), respectively. All procedures were performed according to the steps specified in the kit's instructions.

### RNA isolation and qPCR

Briefly, approximately 50∼80 mg of tissue was taken and mixed with an appropriate amount of TRIzol for homogenization. Following this, chloroform was added, thoroughly mixed, and left for 5 min. After centrifugation at 12,000*g* for 15 min at 4 °C, the supernatant was collected. Isopropyl alcohol was then added, mixed well, and left for 2 h before centrifugation at 12,000*g* and 4 °C for 10 min. The supernatant was discarded, and 80% ethanol (precooled at −20 °C) was added. Centrifugation at 7500*g* for 5 min at 4 °C was done, the supernatant carefully removed, and the pellet dried at room temperature. Subsequently, complementary DNA was synthesized by reverse transcription, and qPCR was performed according to the previous method in our laboratory (22). The primer sequences used for quantitative PCR are listed in Table S3.

### Western blot

Proteins were extracted with Cell lysis buffer for Western and IP and then determined protein concentration using the BCA Protein Assay Kit. Mix the sample with 5% SDS-PAGE protein loading buffer, boil it for 10 min, and separate it on PAGE gel. These separated proteins were then transferred to a polyvinylidene difluoride membrane. The polyvinylidene difluoride membrane was blocked with 5% nonfat milk at room temperature for 2 h. After sealing, incubate the membrane overnight with the first antibody at 4 °C for specific binding. After incubation, wash the membrane three times with TBST (Tris Buffered Saline with Tween-20, Solarbio) and then incubate with the second antibody at room temperature for 2 h. Finally, the bands were visualized and quantified using an ultra-sensitive ECL chemiluminescence detection kit (Beyotime) and Image J software, respectively. β-actin (1:1000, Bioss) expression served as the loading control. The following primary antibodies were used: ATGL (1:1000, Proteintech), ubiquitin (1:1000, Proteintech), HA-tag (1:1000, Proteintech), Flag-tag (1:1000, Proteintech), PPARγ (1:1000, Proteintech).

### Immunoprecipitation

HEK293T cells and grass carp primary adipocytes were cultured on 10 cm dishes. Cells were transfected with the designated plasmids for 48 h, and MG132 was added 6 h before the end. Then, cells were lysed in Cell lysis buffer (P0013, Beyotime) for Western and IP containing protease inhibitors for 30 min and centrifuged at 13,000 g for 15 min. The cell lysates were then incubated with the indicated antibodies and Protein A + G magnetic beads (P2108-1, Beyotime) overnight at 4 °C followed by washing in IP buffer. Immunocomplexes were eluted by boiling at 95 °C for 10 min in SDS-PAGE protein loading buffer. Finally, perform immunoblotting using the indicated primary and corresponding secondary antibodies.

### RNA-seq

Briefly, we isolated adipose tissue from grass carp fed with Control and HFD for 2 weeks and performed RNA-Seq. RNA-seq and analysis were performed by Wuhan Metware Biotechnology Co., Ltd. First, use Trizol to extract total RNA from adipose tissue and detect the RNA quality. Subsequently, library preparation was performed and Agilent 2100 was used to perform quality control of the library. After passing the test, sequencing is performed using the Illumina platform. Genes with fold change > 1.5 and *p*-value < 0.05 were screened as differentially expressed genes. The identified differentially expressed genes were subsequently subjected to KEGG enrichment analyses through the cloud platform.

### Proteomic

#### Protein extraction and digestion

Samples were ground into a fine powder in liquid nitrogen and lysed in buffer containing 7 M urea, 2 M thiourea, 4% SDS, and 40 mM Tris–HCl (pH 8.5), supplemented with 1 mM PMSF and 2 mM EDTA. After reduction with 10 mM DTT and sonication on ice, lysates were centrifuged at 13,000 rpm for 20 min at 4 °C. Proteins in the supernatant were precipitated with four volumes of precooled acetone at −20 °C for 2 h, resuspended in 8 M urea/100 mM TEAB (pH 8.0), reduced with DTT, and alkylated with 50 mM iodoacetamide in the dark. Protein concentration was determined using a BCA assay. Equal amounts of protein (∼100 μg per sample) were digested with trypsin at a 1:50 (w/w) enzyme-to-protein ratio at 37 °C for 12 to 16 h. The resulting peptides were desalted using C18 cartridges and dried by vacuum centrifugation.

#### LC–MS/MS analysis

Liquid chromatography was performed on a nanoElute HPLC (Bruker Daltonics). About 200 ng peptides were separated within 60 min at a flow rate of 0.3 μL/min on a commercially available reverse-phase C18 column with an integrated CaptiveSpray Emitter (25 cm × 75 μm ID, 1.6 μm, Aurora Series with CSI, IonOpticks). The separation temperature was kept by an integrated Toaster column oven at 50 °C. Mobile phases A and B were produced with 0.1 vol % formic acid in water and 0.1% formic acid in ACN. Mobile phase B was increased from 2 to 22% over the first 45 min, increased to 35% over the next 5 min, further increased to 80% over the next 5 min, and then held at 80% for 5 min.

The liquid chromatography was coupled online to a hybrid timsTOF Pro2 (Bruker Daltonics) *via* a CaptiveSpray nano-electrospray ion source (CSI). To establish the applicable acquisition windows for diaPASEF mode, the timsTOF Pro2 was operated in data-dependent parallel accumulation-serial fragmentation (PASEF) mode with 10 PASEF MS/MS frames in one complete frame. The capillary voltage was set to 1400 V, and the MS and MS/MS spectra were acquired from 100 to 1700 m/z. As for ion mobility range (1/K0), 0.7 to 1.4 Vs/cm2 was used. The TIMS accumulation and ramp time were both set to 100 ms, which enable an operation at duty cycles close to 100%. The “target value” of 10,000 was applied to a repeated schedule, and the intensity threshold was set at 2500. The collision energy was ramped linearly as a function of mobility from 59 eV at 1/K0 = 1.6 Vs/cm2 to 20 eV at 1/K0 = 0.6 Vs/cm2. The quadrupole isolation width was set to 2Th for m/z < 700 and 3Th for m/z > 800.

In diaPASEF mode, the instrument control software was extended to define quadrupole isolation windows as a function of the TIMS scan time. Seamless and synchronous ramping of all applied voltage is achieved by modifying the instrument control electronics. We defined 25 Th isolation windows from m/z about 400 to 1200 and totally 64 windows were defined. Other parameters were the same as DDA-PASEF mode.

#### Data processing and quantification

MS raw data were analyzed using DIA-NN(v1.8.1) with library-free method. The C_idella_female_genemodel_caoyu.fasta database (32,811 entries) was used to create a spectra library with deep learning algorithms of neural networks. The option of MBR was employed to create a spectral library from DIA data and then reanalyze using this library. FDR of search results was adjusted to < 1% at both protein and precursor ion levels, the remaining identifications were used for further quantification analysis. This experimental proteomics was completed by Wuhan Metware Biotechnology Co., Ltd. Further analysis of differential proteins was completed using the cloud platform as required by the Wuhan Metware Biotechnology. Identified proteins were analyzed using a significance threshold of *p* ≤ 0.05.

### ChIP-seq analysis

Chromatin immunoprecipitation was performed using standard procedures. Cells or tissues were crosslinked in PBS containing 1% formaldehyde at room temperature for 10 min, and crosslinking was quenched with 125 mM glycine. Samples were washed twice with PBS.

Cells were lysed in lysis buffer (25 mM Tris–HCl, 0.5% SDS, 5 mM EDTA, and protease inhibitors), whereas tissues were homogenized prior to lysis. After incubation on ice for 10 min, chromatin was sonicated to an average size of 100 to 500 bp and immunoprecipitated with the indicated antibodies and magnetic beads overnight at 4 °C. Beads were sequentially washed with low-salt, high-salt, and LiCl buffers, and bound chromatin was eluted.

ChIP DNA libraries were prepared using the NEBNext Ultra DNA Library Prep Kit for Illumina (NEB). End repair/A-tailing, adaptor ligation, and USER enzyme treatment were performed according to the manufacturer’s instructions. Libraries were amplified by 8 to 10 PCR cycles, size-selected, and purified using AMPure XP beads. Library quality was assessed by Qubit and high-sensitivity DNA chip analysis. Libraries were sequenced on an Illumina NovaSeq 6000 platform to generate 150-bp paired-end reads.

Sequencing reads were processed using fastp (v0.20.1) and aligned to the reference genome with Bowtie2 (v2.4.2). Peaks were called using MACS2 (v2.2.7.1), visualized with IGV, and annotated using ChIPseeker. Motif analysis was performed with HOMER (v4.11.1). Differential peak analysis was conducted using MAnorm or DiffBind, as appropriate.

### Dual luciferase reporter assay

The ATGL promoter sequence was cloned into the pGL3.0 basic vector using the ClonExpress Ultra One Step Cloning Kit V3 following the manufacturer's instructions. Subsequently, the JASPAR database (http://jaspar.genereg.net/) was used to predict the HIF1αa-binding sites in the grass carp ATGL promoter. Using a plasmid carrying the ATGL promoter sequence as a template and QuickMutation, the Plus Site Directed Mutagenesis Kit (D0208M, Beyotime) is used for site-directed mutagenesis of HIF1αa-binding sites. Then, plasmids were transfected into HEK293T cells using lipo8000. Finally, ATGL luciferase activity was detected using a dual-luciferase reporter gene assay kit (RG027, Beyotime).

### Statistical analysis

Experimental data were obtained from at least three individual fish from each group. All data are presented as mean ± SD. For comparisons between two groups, a two-tailed Student’s *t* test was performed, Statistical significance is presented below: ∗*p* < 0.05, ∗∗*p* < 0.01, and ∗∗∗*p* < 0.001. For comparisons among three or more groups, one-way ANOVA followed by Tukey’s *post hoc* test was used. Statistical analyses were conducted using SPSS 22.0 software (IBM Corp.,). Differences were considered statistically significant at *p* < 0.05.

## Data availability

The mass spectrometry proteomics data have been deposited to the ProteomeXchange Consortium *via* the iProX partner repository with the dataset identifier PXD072383 (https://proteomecentral.proteomexchange.org). The RNA-seq data have been deposited in the NCBI Sequence Read Archive (SRA) under accession number PRJNA1393194 and PRJNA1393244. The ChIP-seq data have been deposited at GEO and are publicly available under accession number GSE315218 at https://www.ncbi.nlm.nih.gov/geo/query/acc.cgi?acc=GSE315218.

## Supporting information

This article contains supporting information.

## Conflict of interest

The authors declare that they have no conflicts of interest with the contents of this article.
