# Supplementary material for: Hypoxia-inducible factor 1αa regulates lipid metabolism to coordinate adipocyte hypertrophy and hyperplasia in grass carp
Source: J Biol Chem. 2026 Jan 27;302(3):111195. doi: 10.1016/j.jbc.2026.111195 (PMC12930053; doi:10.1016/j.jbc.2026.111195)
Supplement: Table S3 [file mmc3.docx]

**Table S3. Primers used in the qPCR analysis.**

| Gene name | Sense and antisense primer (5’–3’) | GenBank no. |
| --- | --- | --- |
| β-actin | F: TCCACCTTCCAGCAGATGTGGATT  R: AGTTTGAGTCGGCGTGAAGTGGTA | DQ211096.1 |
| PPARγ | F: CGCTCATCTCCTACGGTCAG  R: ATGTCGCTGTCGTCCAACTC | EU847421.1 |
| DGAT2 | F: CACCTTCCAAGTACCTTCTG  R: AGATCCCACTCGCCTATT | XM_051907885.1 |
| ATGL | F: TCGTGCAAGCGTGTATATG  R: GCTCGTACTGAGGCAAATTA | HQ845211.2 |
| IL-1β | F: GCCAAGTAGCCGAATCACAGA  R: AAGCCCAAGATATGCAGGAGTC | MK942107.1 |
| IL-8 | F: ATGAGTCTTAGAGGTCTGGGT  R: ACAGTGAGGGCTAGGAGGG | JN663841.1 |
| TNFα | F: CGCTGCTGTCTGCTTCAC  R: CCTGGTCCTGGTTCACTC | HQ696609.1 |
| HIF1αa | F: CAGATGGTGTTGTGAAGGGTG  R: GTGGCGGCTGAGGAACG | [MK576005.1](https://www.ncbi.nlm.nih.gov/nuccore/1743538684) |
| VEGFα | F: AGCGTTGAAAGACAGAGCGA  R: AACGGCTTCTGTGCGTATGA | XM_051864921 |
| ANGPTL4 | F: TTCTGCGAAATGACACCCGA  R: CCGAGCCAGAACTCACCATT | XM_051881331 |
| TGFβ | F: TTGGGACTTGTGCTCTAT  R: AGTTCTGCTGGGATGTTT | EU099588 |

PPARγ, [peroxisome proliferator-activated receptor](https://www.citexs.com/allSearchDetail?wid=4210318371) γ; DGAT2, diacylglycerol acyltransferase 2; ATGL, [adipose triglyceride lipase](https://kns.cnki.net/kns8/Detail/RedirectScholar?flag=TitleLink&tablename=GARJ2021_3&filename=SJES908C0EB75AEEE6FC28279070A6C1455F); IL-1β, interleukin-1β; IL-8, interleukin-8; TNFα, tumor necrosis factor α; HIF1αa, hypoxia inducible factor-1αa; VEGFα, vascular endothelial growth factor α; ANGPTL4, Angiopoietin-like Protein 4; TGFβ, Transforming growth factor β.
