# Supplementary material for: Hypoxia-inducible factor 1αa regulates lipid metabolism to coordinate adipocyte hypertrophy and hyperplasia in grass carp
Source: J Biol Chem. 2026 Jan 27;302(3):111195. doi: 10.1016/j.jbc.2026.111195 (PMC12930053; doi:10.1016/j.jbc.2026.111195)
Supplement: Table S4 [file mmc4.docx]

**Table S4. Key resources table.**

| REAGENT or RESOURCE | SOURCE | IDENTIFIER |
| --- | --- | --- |
| Antibodies | | |
| Rabbit polyclonal anti ATGL | Proteintech | Cat#: 55190-1-AP |
| Rabbit polyclonal anti ubiquitin | Proteintech | Cat#: 10201-2-AP |
| Rabbit polyclonal anti HA tag | Proteintech | Cat#: 51064-2-AP |
| Rabbit polyclonal anti Flag tag | Proteintech | Cat#: 20543-1-AP |
| Rabbit polyclonal anti PPARγ | Proteintech | Cat#: 16643-1-AP |
| Rabbit polyclonal anti beta-actin | Bioss | Cat#: bs-0061R |
| Goat anti-rabbit IgG (H&L) | Deeyee | Cat#: DY60202 |
| Goat anti-rabbit IgG (H&L) FITC | Proteintech | Cat#: SA00003-2 |
| Rabbit polyclonal anti Caveolin-1 | Proteintech | Cat#: 16447-1-AP |
| Rabbit polyclonal anti F4/80 | Bioss | Cat#: bs-11182R |
| Bacterial and virus strains | | |
| *Escherichia coli* DH5α | Thermo Fisher | Cat#: EC0112 |
| Biological samples |  |  |
| Grass carp adipose tissue | This paper | N/A |
| Chemicals, peptides, and recombinant proteins | | |
| DAPI | Beyotime | Cat#: C1002 |
| DMSO | MCE | Cat#: HY-Y0320 |
| CoCl_2_ | Sigma-Aldrich | Cat#: 409332 |
| GW9662 | MCE | Cat#: HY-16578 |
| PX-478 | MCE | Cat#: [HY-10231](https://www.medchemexpress.cn/PX-478.html) |
| MG132 | MCE | Cat#: [HY-13259](https://www.medchemexpress.cn/MG-132.html) |
| EdU | MCE | Cat#: HY-118411 |
| Bodipy | Beyotime | Cat#: C2053S |
| BODIPY™ 558/568 C12 | Thermo Fisher | Cat#: D3835 |
| Lipo8000 | Beyotime | Cat#: C0533 |
| Cell lysis buffer for Western and IP | Beyotime | Cat#: P0013 |
| Ultra-sensitive ECL chemiluminescence detection kit. | [Epizyme](mailto:info@epizyme.cn) | Cat#: SQ201 |
| Protein A+G magnetic beads | Beyotime | Cat#: P2108-1 |
| SDS-PAGE protein loading buffer | Beyotime | Cat#: P0015L |
| Critical commercial assays | | |
| Tissue cell triglyceride (TG) enzymatic assay kit | Applygen | Cat#: E1013 |
| Free fatty acid (FFA) content assay kit | Solarbio | Cat#: BC0595 |
| BeyoClick™ EdU-555 Cell Proliferation Detection Kit | Beyotime | Cat#: C0075L |
| Hypoxia probe detection kit | Hypoxyprobe | Cat#: HP1-100Kit |
| ClonExpress Ultra One Step Cloning Kit V3 | Vazyme | Cat#: C117-01 |
| QuickMutation™Plus Site-Directed Mutagenesis Kit | Beyotime | Cat#: D0208M |
| BCA Protein Assay Kit | Solarbio | Cat#: PC0020 |
| Double luciferase reporter gene test kit | Beyotime | Cat#: RG027 |
| Deposited data | | |
| Adipose tissue RNA-seq data | This paper | PRJNA1393244 |
| Adipocyte RNA-seq data | This paper | PRJNA1393194 |
| Adipose tissue Proteomics date | This paper | PXD072383 |
| Adipocyte PPARγ ChIP-seq date | This paper | GSE315218 |
| Experimental models: Cell lines | | |
| HEK293T | Beyotime | Cat#: C6008 |
| Grass carp adipocyte | This paper | N/A |
| Experimental models: Organisms/strains | | |
| Grass carp | Ankang Fishery Experimental Demonstration Station. | N/A |
| Recombinant DNA | | |
| pCDN3.1-HA-ATGL | This paper | N/A |
| pEGFP-N1-HIF-1αa | This paper | N/A |
| pCMV-Flag-ubiquitin | This paper | N/A |
| pPPARγ-TA-Luc | Beyotime | Cat#: D4288 |
| Software and algorithms | | |
| Image J | National Institutes of Health | [https://imagej.nih.gov/ij/](https://imagej.nih.gov/ij/) |
| Graphpad Prism | Graphpad | [https://www.graphpad.com/](https://www.graphpad.com/) |
| Other | | |
| Grass carp high-fat diet | This paper | N/A |
