# Supplementary material for: Hypoxia-inducible factor 1αa regulates lipid metabolism to coordinate adipocyte hypertrophy and hyperplasia in grass carp
Source: J Biol Chem. 2026 Jan 27;302(3):111195. doi: 10.1016/j.jbc.2026.111195 (PMC12930053; doi:10.1016/j.jbc.2026.111195)
Supplement: Supplementary Data [file mmc5.docx]

**SUPPORTING INFORMATION**

Hypoxia-Inducible Factor 1αa Regulates Lipid Metabolism to Coordinate Adipocyte Hypertrophy and Hyperplasia in Grass Carp

Mingkui Wei, Zhiqi Tian, Lei Song, Rongrong Xue, Handong Li, Hong Ji, and Jian Sun*

College of Animal Science and Technology, Northwest A&F University, Yangling, China

*Corresponding author: [sunnjjian@nwsuaf.edu.cn](mailto:sunnjjian@nwsuaf.edu.cn)

**Figure S1. Frequency distribution of adipocyte area in grass carp**

**
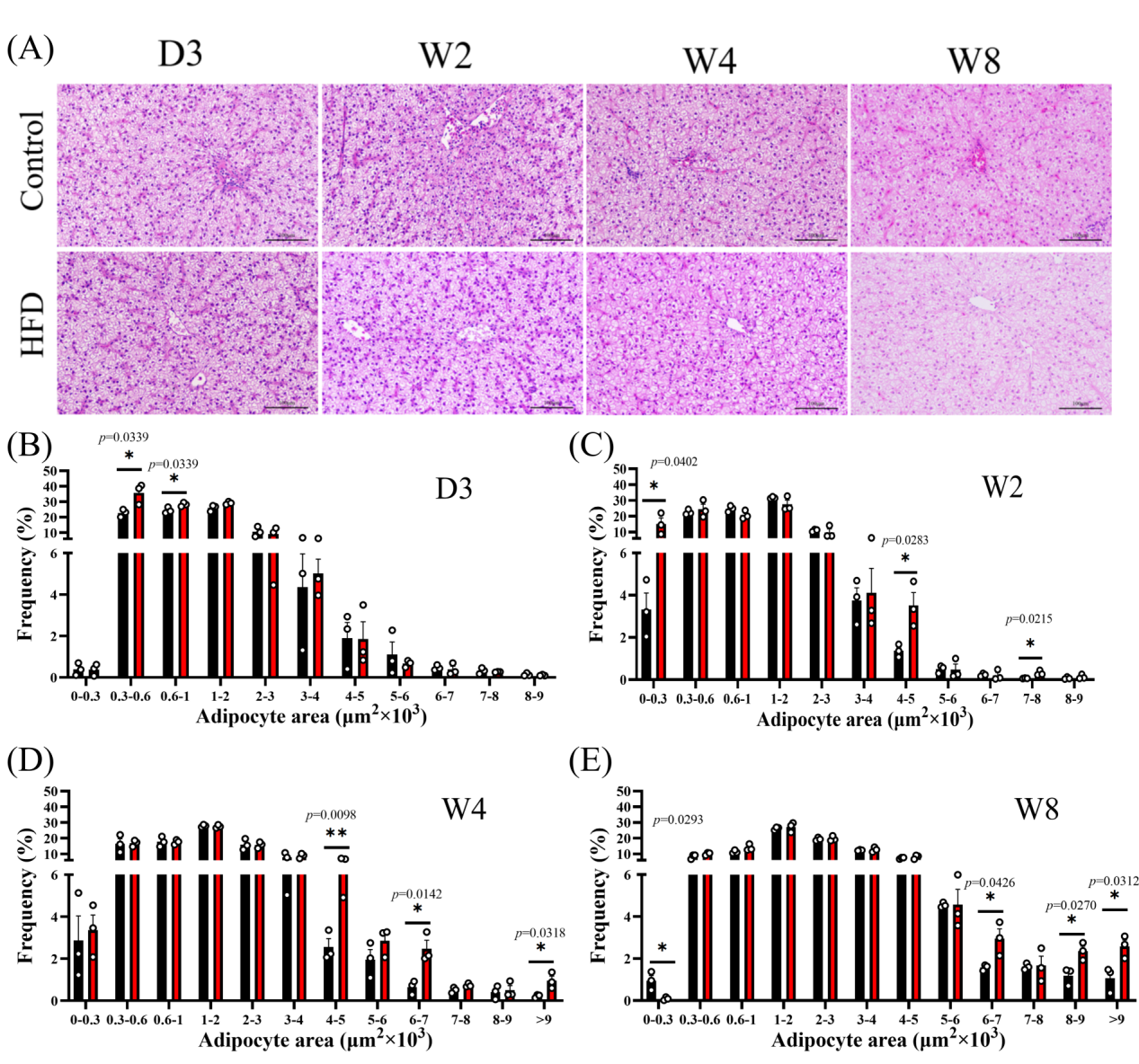
**

1. H&E stains of the liver from grass carp fed Control and HFD for 3 days (D3), 2 weeks (W2), 4 weeks (W4), and 8 weeks (W8). Scale bar 50 μm.
2. E) Frequency distribution of adipocyte area.

Results are presented as means ± SD. Data analysis was conducted by Student’s two-tailed t test. Results with *p* < 0.05 represents a statistically significant difference: **p* < 0.05; ***p* < 0.01; ****p* < 0.001.

**Figure S2. Long-term HFD (W8) activates downstream target genes of HIF1αa.**

**
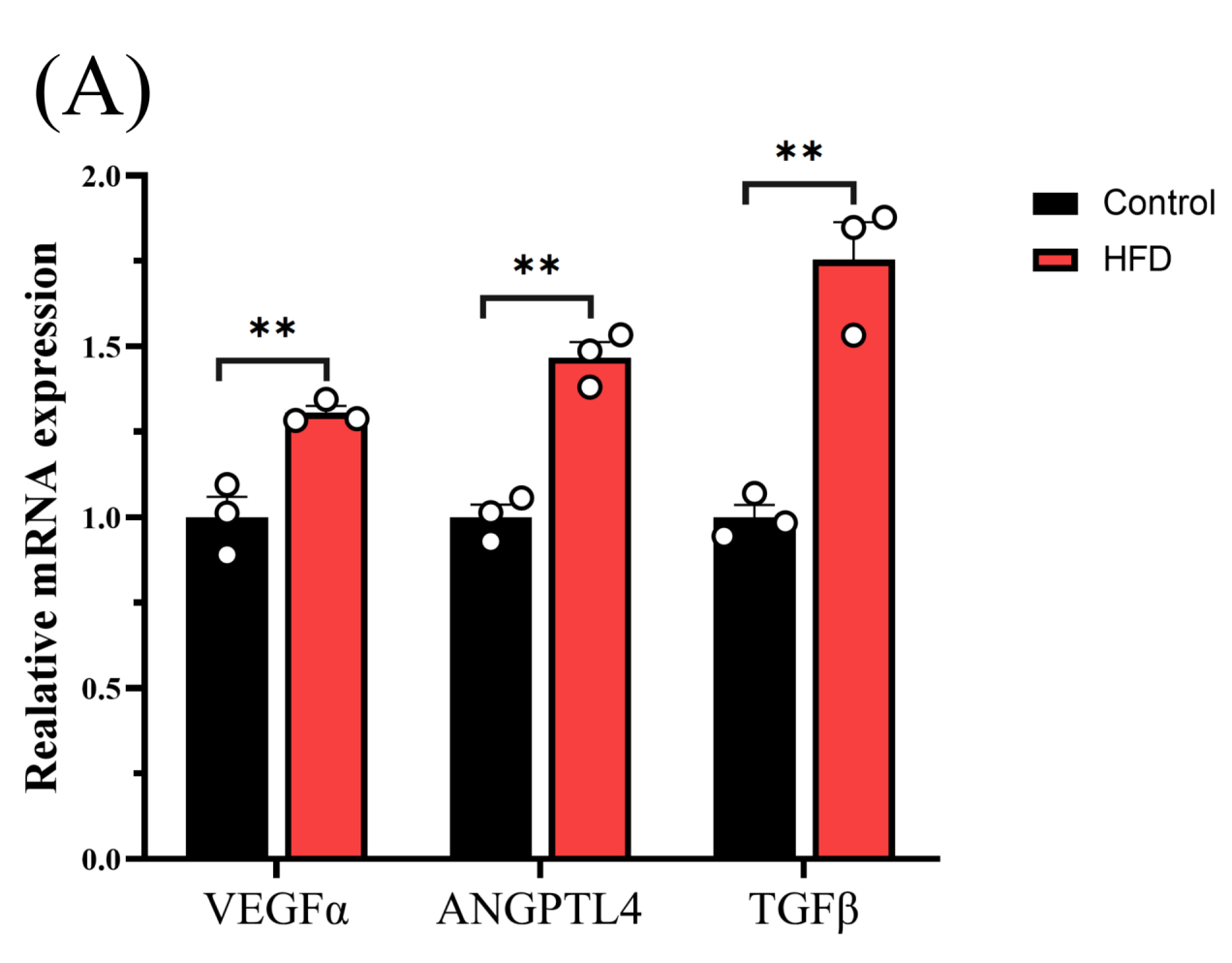
**

1. The mRNA expression of HIF1αa downstream target genes VEGFα, ANGPTL4, TGFβ in the adipose tissue of grass carp after 8 weeks of Control and HFD feeding, n=3. Results are presented as means ± SD. Data analysis was conducted by Student’s two-tailed t test. Results with *p* < 0.05 represents a statistically significant difference: **p* < 0.05; ***p* < 0.01; ****p* < 0.001.

**Figure S3. ChIP-seq analysis of PPARγ binding in co-cultured preadipocytes.**

**
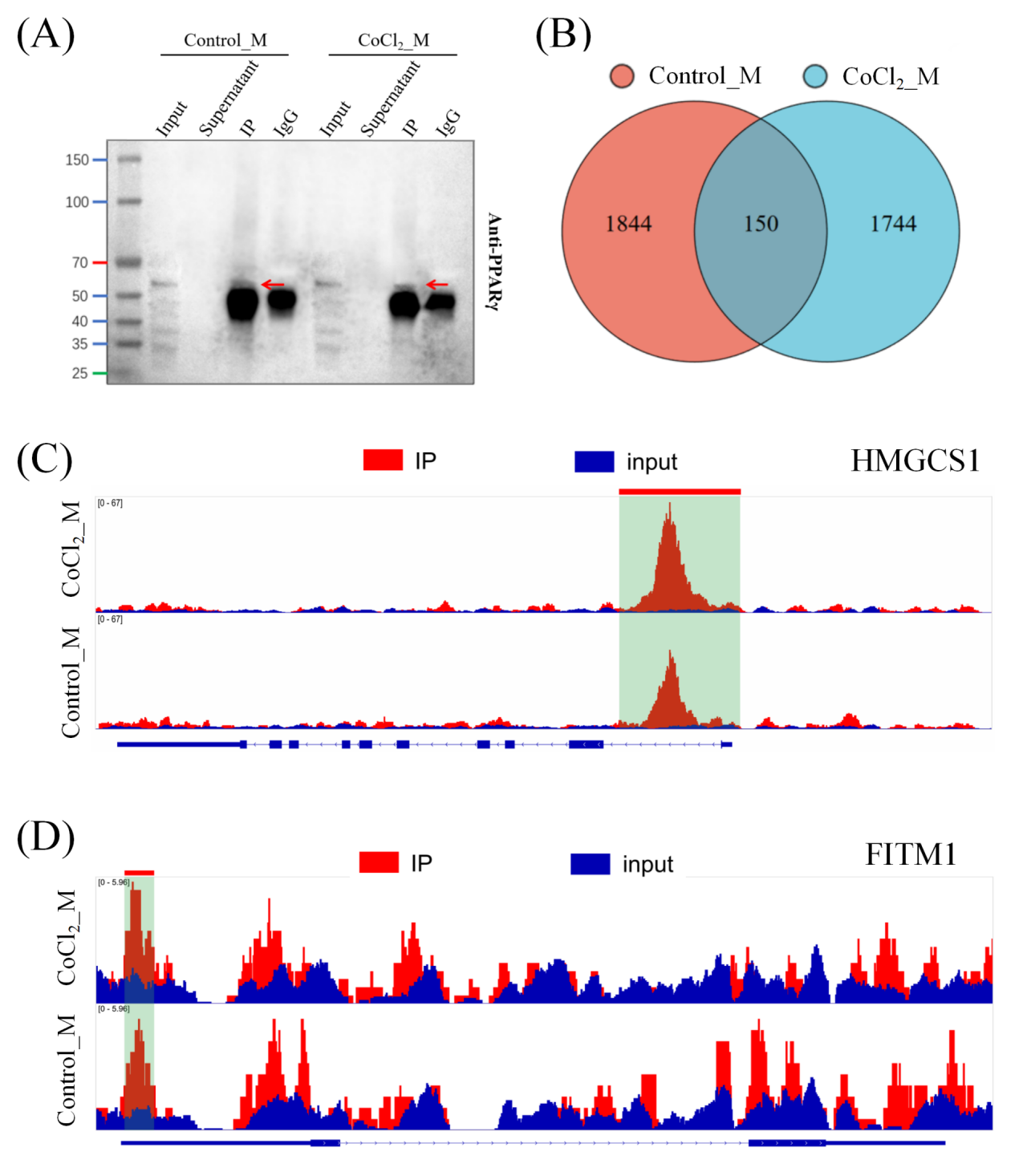
**

1. Validation of PPARγ immunoprecipitation efficiency by Western blot. Red arrows indicate the specific PPARγ bands.
2. Venn diagram of PPARγ ChIP-seq peaks within 4.5 kb of predicted transcription start sites.
3. Examples of PPARγ ChIP-seq peaks on known lipogenesis target genes HMGCS1.
4. Examples of PPARγ ChIP-seq peaks on known lipogenesis target genes FITM1.
